# Metformin and propranolol combination prevents cancer progression and metastasis in different breast cancer models

**DOI:** 10.18632/oncotarget.13760

**Published:** 2016-12-01

**Authors:** María Rico, María Baglioni, Maryna Bondarenko, Nahuel Cesatti Laluce, Viviana Rozados, André Nicolas, Manon Carré, O. Graciela Scharovsky, Mauricio Menacho Márquez

**Affiliations:** ^1^ Instituto de Genética Experimental, Facultad de Ciencias Médicas, Universidad Nacional de Rosario, Rosario, Argentina; ^2^ El Consejo Nacional de Investigaciones Científicas y Técnicas, Argentina; ^3^ Aix-Marseille Université, Inserm UMR_S 911, Centre de Recherche en Oncologie biologique et Oncopharmacologie, Faculté de Pharmacie, Marseille, France; ^4^ Service d'Hématologie and Oncologie Pédiatrique, AP-HM, Marseille, France; ^5^ Metronomics Global Health Initiative, Marseille, France

**Keywords:** breast cancer, metronomics, drug repositioning, metformin, propranolol

## Abstract

Discovery of new drugs for cancer treatment is an expensive and time-consuming process and the percentage of drugs reaching the clinic remains quite low.

Drug repositioning refers to the identification and development of new uses for existing drugs and represents an alternative drug development strategy.

In this work, we evaluated the antitumor effect of metronomic treatment with a combination of two repositioned drugs, metformin and propranolol, in triple negative breast cancer models.

By in vitro studies with five different breast cancer derived cells, we observed that combined treatment decreased proliferation (*P* < 0.001), mitochondrial activity (*P* < 0.001), migration (*P* < 0.001) and invasion (*P* < 0.001). *In vivo* studies in immunocompetent mice confirmed the potential of this combination in reducing tumor growth (*P* < 0.001) and preventing metastasis (*P* < 0.05).

Taken together our results suggest that metformin plus propranolol combined treatment might be beneficial for triple negative breast cancer control, with no symptoms of toxicity.

## INTRODUCTION

Among breast cancer subtypes, triple-negative breast cancer (TNBC) exhibit characteristics distinct from the others, as they are particularly aggressive, frequently recur and become metastatic. TNBC accounts for 15% of all breast cancer types with higher percentages in premenopausal African-American and Hispanic women, and it is associated with very poor prognosis and limited treatment options availability [1].

Drug repositioning refers to the assignation of new uses for existing drugs and represents an alternative drug development strategy. In oncology there is an increasing interest in the use of non-cancer drugs for cancer treatments due to the previous detailed knowledge of pharmacokinetics/dynamics and toxicities, and because most repositioned drugs are available at low cost, normally as generics [2] which provides an opportunity to bypass partially the early costs and time associated to new drugs development. This is a particular issue for low-income countries, where the availability of drugs for cancer treatment is very limited and restricted to some essential cytotoxic drugs and cost is a major factor in influencing access to cancer therapies [3].

Drug repositioning is frequently combined with metronomic chemotherapy to what has been defined as “metronomics” [3]. Metronomic chemotherapy refers to the regular administration of conventional chemotherapy drugs at low, minimally toxic doses, without long resting periods of time [4]. Importantly, several phase II trials have shown effectiveness of metronomic therapies on different cancer types including TNBC with different drugs [5]. Interestingly, developed and approved drugs that showed an anti-cancer opportunity for therapy can be administered orally, on daily basis, in a metronomic fashion.

The anti-diabetic metformin (Met) has been shown to have anticancer properties involving both direct (insulin-independent) and indirect (insulin-dependent) actions. Retrospective studies have reported that patients with diabetes receiving Met exhibited decreased cancer incidence and cancer-related mortality [6–8]. These studies were complemented with a growth-inhibitory effect observed for Met on breast cancer cells in culture and a reduction on mammary tumor growth in mice [9, 10] and a synergistic action of Met with EGFR inhibitors [11, 12]. Interestingly, Met was also found to be effective against tumors expressing constitutively active phosphatidylinositol-3 kinase [PI3K; 13] and trastuzumab-refractory breast cancer xenografts [14]. Also, preliminary results from clinical trials showed that Met administration have an impact on tumor proliferation markers [15].

The indirect anticancer effect of Met involves insulin dependent actions, associated with a reduction of insulin circulating levels that lead to a decrease in the mitogenic and antiapoptotic potential of insulin. In this way, Met may diminish the pro-stimulatory effect of insulin on cancer cells. Met direct effects are linked to inhibition of mitochondrial complex I [16]. This inhibition interrupts mitochondrial respiration, decreasing proton-driven synthesis of ATP, causing cellular energetic stress and elevation of the AMP:ATP ratio which, in turn, activates AMP-activated protein kinase (AMPK), a key cellular energy sensor kinase [10]. AMPK activation leads to a reduction in mammalian target of rapamycin (mTOR) signaling, protein synthesis and proliferation [16–18].

Propranolol (Prop) is a noncardioselective β-adrenergic receptor blocker with reported antioxidant and anti-inflammatory properties, used traditionally for hypertension, angina pectoris, myocardial infarction, migraines, anxiety disorders, and tremor [19]. It was previously shown that Prop reduces intracellular calcium levels, Bax-mediated cytochrome C release and inhibits protein kinase C (PKC) activity in a β-adrenoreceptor independent manner [19–21], and it induces cell cycle arrest and apoptosis via Akt/MAPK pathway in melanoma cells [22]. Many studies in humans have demonstrated its efficacy for the treatment of infantile haemangioma. In this regard, it seems that Prop exerts its suppressive effects acting through the HIF-1α-VEGF-A angiogenesis axis, with effects mediated through the PI3K/Akt and p38/MAPK pathways [23]. In relation to breast cancer, retrospective studies reported an improved survival with reduction in the risk of recurrence in woman receiving this β-blocker therapy [24].

In this study we evaluated the effect of metronomic combined administration of two repositioned drugs, Met and Prop, on different models of TNBC both *in vitro* and *in vivo*. We found that combined treatment was effective on preventing cell growth, triggering apoptosis and decreasing migratory/invasive capabilities of cells *in vitro*, probably through their action on mitochondrial bioenergetics. In a similar way, combined treatment was able to reduce tumor growth *in vivo*, preventing development of lung metastasis and increasing mice survival, without symptoms of toxicity. These results unveil a new combinatorial low-cost treatment with no signs of toxicity for a type of tumors that normally have limited treatment options paving the way for genuine global oncology.

## RESULTS

### Met and Prop act synergically on breast tumor cell lines viability

**Figure 1:**
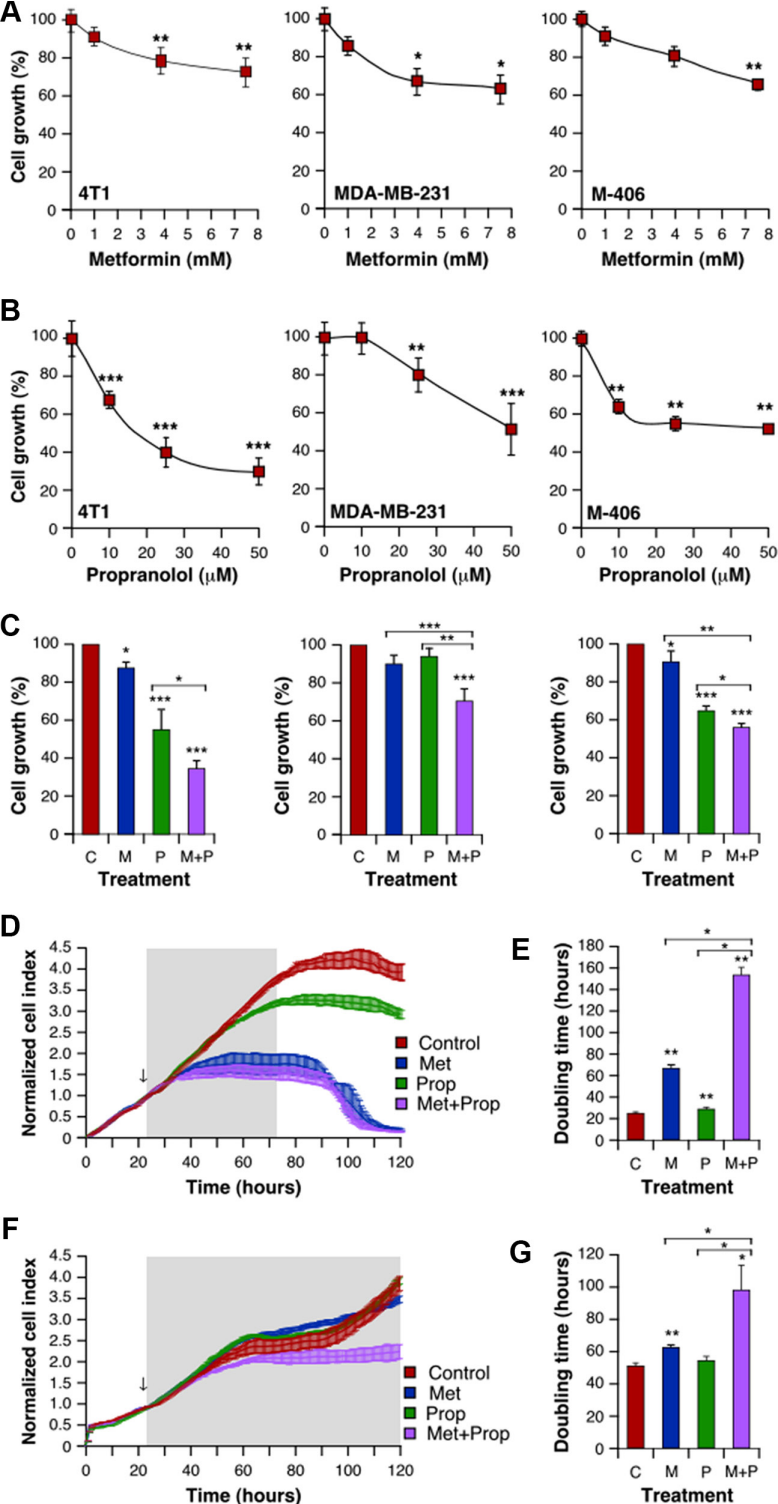
Met and Prop effect on breast tumor cells viability Cells were cultured in the presence of the indicated doses of Met (**A**) or Prop (**B**) during 24 hours. The number of metabolically active cells was estimated by tetrazolium salts reduction method (*n* = 3). (**C**) 4T1 (left panel), MDA-MB-231 (middle panel) and M-406-derived cells (right panel) were treated for 24 hours with Met (M; 1 mM), Prop (P; 1 μM) or a combination of them (M+P) and living cells were estimated as before (*n* = 3). (**D**–**G**) Cell proliferation and doubling time evaluation by real time impedance-based method for 4T1 (**D**, **E**) and MDA-MB-231 (**F**, **G**). Arrows indicate the moment of treatment addition (Met 1 mM, Prop 10 μM).

We first analyzed the effect of treatment with Met and Prop on the growth of 5 different breast tumor-derived cells. Both drugs were able to reduce cell growth in a dose dependent manner (Figure 1A, 1B and Supplementary Figure S1A, S1B) at different levels in all the cells tested, as seen by colorimetric assays. Noteworthy, combined treatment with Met + Prop showed a stronger effect on cell growth that single agent treatment (Figure 1C and Supplementary Figure S1C). Interestingly, nor single or combined treatments caused significant effects on non-tumoral cell line MDCK (Supplementary Figure S1A–S1C). Dynamic monitoring of cell proliferation using impedance technology strengthened these observations. 4T1 cells growth kinetics was slowed down by Met or Prop individual treatment compared to vehicle-treated cells (Figure 1D). Indeed, cell-doubling time determined before cell confluence, was significantly (Met: *P* < 0.05; Prop: *P* < 0.01) increased by those treatments (Figure 1E). More importantly, combination of Met and Prop was significantly more potent to reduce ability of 4T1 cells to divide, showed by growth kinetics and doubling time calculation (Figure 1D, 1E; Supplementary Figure S1D). Drug cytostatic effects were further highlighted by 4T1 cell population stabilization from 16 h after starting treatment (i.e. 40–88 h; Figure 1D). Combining Met with Prop led to greater activity as shown by reductions in slope values (Supplementary Figure S1E). Impedance measurements in MDA-MB-231 cells confirmed that combination of Met and Prop was effective leading to a decrease in both growth kinetics and doubling time (Figure 1F, 1G). Indeed, the combination was found to act synergically on 4T1 and MDA-MB-231 cells, as demonstrated by calculation of IC_50_ for each drug in the presence of the other (Supplementary Figure S1F, S1G).

As Met and Prop administration to patients is usually carried out on a “metronomic” manner, we decided to explore if the continuous exposure to these drugs could lead to stronger effects on cell proliferation. We compared the impact on proliferation of short-term treatment (approximately equivalent to a cell doubling time) and a continuous 144 hours metronomic treatment. Calculations of IC_50_ in these two conditions revealed that treatment with Met and Prop in a metronomic basis increased drastically (as example, Met IC_50_ values for 4T1 cells: from 5.8664 to 0.1656) cells sensitivity to these two repositioned drugs (Table 1).

**Table 1:** IC_50_ values of metformin and propranolol on 4T1, MDA-MB-231 and MCF7 cells treated for 36 h or 144 h

Cell type	Short term treatment				Metronomic treatment			
	IC_50_Met(mM)	SEM	IC_50_Prop(μM)	SEM	IC_50_Met(mM)	SEM	IC_50_Prop(μM)	SEM
4T1	5.8664	0.2318	5.2007	0.4317	0.1656	0.0079	0.1042	0.0154
MDA-MB-231	5.9028	0.3928	7.9109	0.2006	0.2742	0.0112	0.2148	0.0091
MCF7	1.0799	0.0701	7.9716	0.1954	0.0709	0.0283	0.1788	0.0262

Results are expressed as mean ± SEM. In all the cases for each drug and cell type, IC_50_ (short term treatment) vs IC_50_(metronomic treatment) *P* < 0.001.

**Figure 2:**
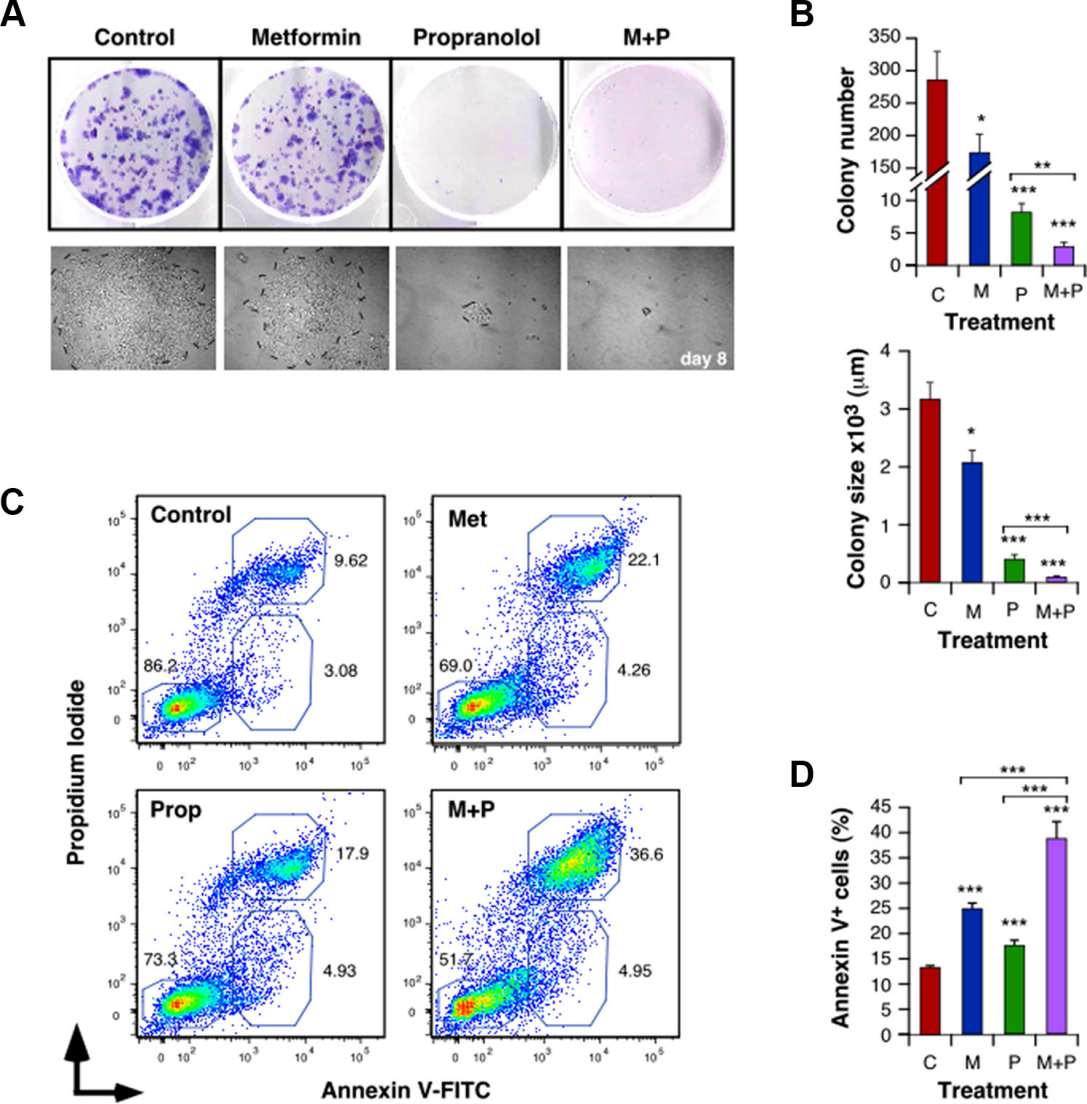
Met and Prop affect the clonogenic behaviour and trigger apoptosis in breast cancer cells Cells (500 cells/well) were cultured in the presence of Met (5 mM) and/or Prop (5 μM) during 8 days. Colonies were visualized by Giemsa staining (**A**) in order to allow quantification (**B**, top panel). Clones photos were taken at different times (**A**, lower panel) and their size was estimated by measuring colonies diameters with the Image J software (**B**, bottom panel). (**C**, **D**) Analysis of the degree of apoptosis triggered by Met (5 mM) and/or Prop (5 μM): After 24 hours of treatment with the indicated drugs, 4T1 cells were collected, washed and stained with Annexin V-FITC and Propidium Iiodide. The percentage of apoptotic populations was analyzed by flow cytometry. (**C**) Flow cytomety profiles for 4T1 cells. (**D**) Quantification of the percentage of Annexin V^+^ apoptotic cells. (M: Met, P: Prop, M+P: Met+Prop; *n* = 3).

Treatment with Met and Prop also affected the ability of 4T1 (Figure 2A, 2B and Supplementary Figure S2A) and MDA-MB-231 (Supplementary Figure S2B) cells to form colonies, altering the number of viable clones (Met+Prop, *P* < 0.001) and their size (Met + Prop, *P* < 0.001).

To further characterize the effect of Met and Prop on viability, we evaluated the induction of apoptosis by treatments. Both, Met and Prop increased significantly the number of 4T1 and MCF7 apoptotic cells (Figure 2C, 2D and Supplementary Figure S2C). Prop induced apoptosis in MDA-MB-231 and M-234p-derived cells. In a similar manner, Met alone promoted increase in apoptotic population of M-406-derived cells (Supplementary Figure S2C). Nevertheless, regardless the cell type or the individual treatment effect, combination of drugs triggered apoptotic response in all the cells tested, in significant higher levels than any individual treatment (Figure 2C, 2D and Supplementary Figure S2C).

### Combining Met to Prop leads to a strong inhibition of mitochondrial bioenergetics

**Figure 3:**
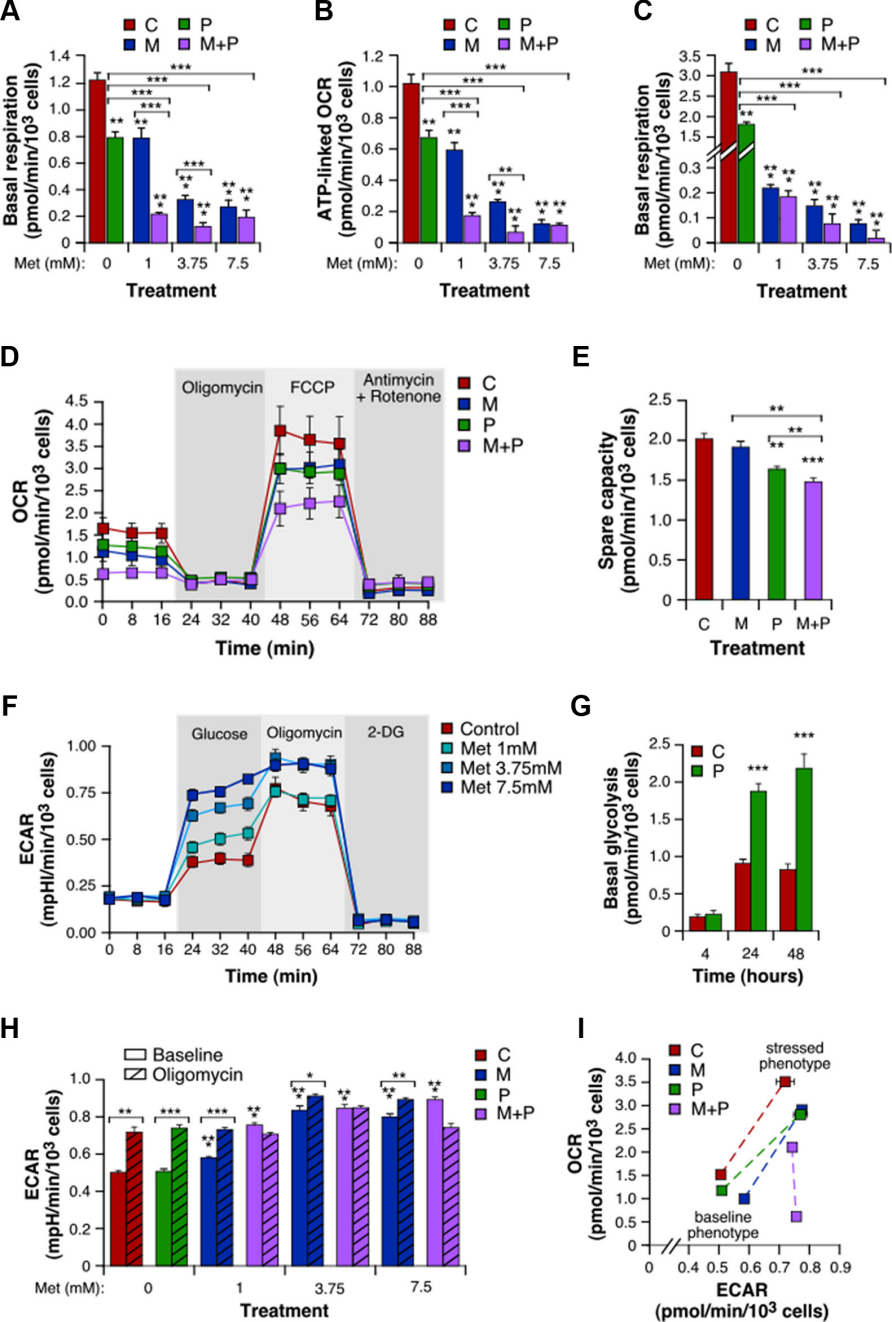
Combination of Met and Prop shows anti-mitochondrial properties and stimulates glycolysis 4T1 cells treated with Prop (10 μM) and Met (1, 3.75 and 7.5 mM) alone or in combination for 4 h were analyzed for mitochondrial bioenergetics using the Seahorse XF technology. Measurement of basal level of oxygen consumption rate (OCR) was followed by sequential injections of FCCP and rotenone/antimycin A to allow determination of the amount of non-ATP-linked oxygen consumption (proton leak), the maximal respiration and the non-mitochondrial oxygen consumption. (**A**) Basal respiration, calculated as the difference between basal OCR and non-mitochondrial OCR. (**B**) ATP-linked OCR, defined as the difference between basal OCR and non-ATP-linked oxygen consumption. (**C**) Basal respiration of 4T1 cells exposed to Met and Prop for 24 h. (**D**) Real-time mitochondrial bioenergetic profile of live 4T1 cells after a 4 h-treatment with Prop 10 μM, Met 1 mM, and the combination. (**E**) Spare capacity, defined as the difference between basal and maximal rates. (**F**) Real-time measure of extracellular acidification rate (ECAR) was performed in intact 4T1 cell cultures first incubated in assay medium without glucose and pyruvate, and then successively supplemented with glucose, with the ATP synthase inhibitor oligomycin that consequently accelerate the glycolytic process, and with the glycolysis inhibitor 2-deoxy-glucose (2-DG). (**G**) Basal glycolysis, calculated as the difference between glucose-mediated ECAR and rates without glucose, in 4T1 cells treated with vehicle or Prop 10 μM for the indicated times. (**H**) Basal and oligomycin-stimulated ECAR in cells incubated for 4 h with a range of concentrations of drugs alone or combined. (**I**) Energy phenotype of 4T1 cells after a 4 h-treatment with Prop and/or Met. Mitochondrial respiration and glycolysis were first simultaneously analyzed under the starting medium conditions (Baseline Phenotype) and then upon injection of FCCP and oligomycin, respectively (Stressed Phenotype). The metabolic potential (dotted lines) revealed 4T1 cells ability to meet an energy demand, and preferred pathway. (M: Met, P: Prop, M + P: Met + Prop; *n* = 3).

To assess the effect of treatment on mitochondrial respiration in intact 4T1 cells, we performed real-time measurements of the oxygen consumption rate (OCR). After a 4 h-incubation with Prop, cells showed a basal respiration and a mitochondria-governed ATP generation reduced by 35% and 33%, respectively, as compared to control (Figure 3A, 3B). Met also induced a noticeable dose-dependent decrease in both mitochondrial respiration and ATP production, which reached -77% and -83% respectively at 7.5 mM (Figure 3A, 3B). These mitochondrial activities were completely abolished at 24 h, even with the lowest concentration of Met (-93% respiration at 1 mM; Figure 3C). Importantly, the mitochondrial inhibitory functions of Met were increased by Prop (Figure 3A–3C). This was especially the case for the lowest concentration of Met that, when combined to Prop, further significantly reduced basal respiration by 71% (Figure 3A) and respiration-linked ATP by 69% (Figure 3B). When electron transport chain uncoupler FCCP was added, mitochondrial respiration was stimulated mimicking an increase in energy demand and showing the maximal respiration rate (Figure 3D). In this case, the combination between small doses of Met with Prop was still more active than the two drugs alone to reduce 4T1 cells oxygen consumption rate (OCR). In line with this, the spare capacity –defined as the difference between maximal and basal respiration– was also significantly diminished by the combinatorial treatment (*P* < 0.01; Figure 3E). These results definitely demonstrated the potent anti-mitochondrial properties of the Prop/Met combination, even at low concentrations.

### Met and Prop combination drastically activates glycolysis

Cancer cells are known for their ability to shift their metabolic phenotype. Taking into account the inhibition of the mitochondrial oxidative phosphorylation by Prop and Met, we then determined whether 4T1 cells in turn hyper-activated glycolysis. We therefore measured the extracellular acidification rate (ECAR) that reflects lactate release over time. In this context, we found that cells exposed to different doses of Met for 4 h showed a dose-dependent increase in ECAR, after glucose addition (Figure 3F). Prop treatment also stimulated basal glycolysis, in a time-dependent manner (Figure 3G). While its effect was limited after 4 h when used alone, Prop significantly enhanced the pro-glycolysis properties of Met, especially for the lowest concentrations (Figure 3H). Following addition of the ATP synthase inhibitor oligomycin, cells treated with either vehicle, Prop alone or Met alone exhibited higher ECAR (Figure 3H). In contrast, the Met/Prop combinations –whatever the concentrations used– forced 4T1 cells to activate glycolysis up to the maximum extent, as revealed by their insensitivity to oligomycin. Lastly, we represented 4T1 cell energy phenotype that comprises a baseline phenotype (starting medium condition), a stressed phenotype (mediated by FCCP for OCR and by oligomycin for ECAR), and a metabolic potential (Figure 3I). This last parameter reveals the cells’ ability, and preferred energetic pathway, to respond to supplemental energy demand due to stress. It confirmed that, when exposed to the combinatorial treatment, 4T1 cells energy phenotype dramatically changed, due to both treatment inhibitory properties of mitochondria oxidative phosphorylation and cells incapacity to further mobilize the glycolytic pathway.

### Met and Prop affect metastasis related events *in vitro*

**Figure 4:**
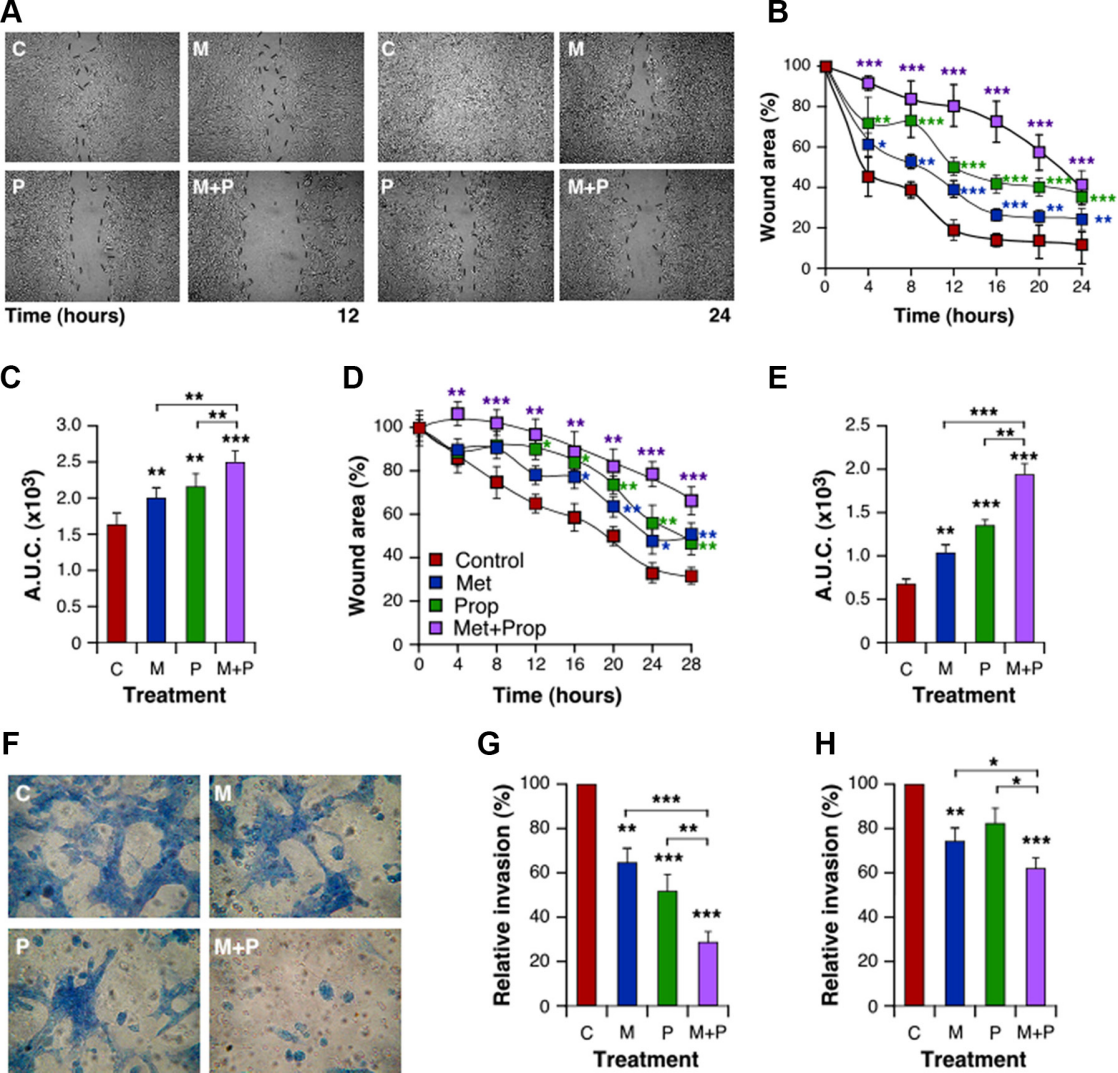
Met and Prop affect metastatic-related events in vitro Wound healing assay was performed as described in Materials and Methods. Cellular motility was estimated by measuring closure of the initial wound. Photos were taken at the indicated times (**A**, 4T1 cells). Quantification of healing was performed using the Image J software and the area under the curve (A.U.C.) was calculated (**B**, **C**; 4T1 cells; **D**, **E**: MDA-MB-231 cells; *n* = 3). (**F**–**G**) Cells were seeded onto matrigel-coated transwells and incubated in presence or absence of drugs. Invading cells were stained with Giemsa (**F**). Quantification of invading cells was performed for 4T1 (**G**) and MDA-MB-231 (**H**) cells. Met 5 mM; Prop 5 μM.

In order to characterize properties of the cells related to metastatic processes, we explored breast cancer cell migration and invasion abilities in the presence of Met and/or Prop. Both drugs significantly reduced (*P* < 0.01) the migratory properties of 4T1 and MDA-MB-231 cells in an *in vitro* wound-healing assay (Figure 4A–4E). In addition, both drugs reduced the invasion efficiency of 4T1 and MDA-MB-231 cells in a conventional assay with matrigel, but only metformin did it significantly (*P* < 0.01) for human cells (Figure 4F–4H). Furthermore, as previously shown for growth and apoptosis, combined treatment exacerbated migratory and invasive defects triggered by treatments in both cell lines (Figure 4A–4F, Supplementary Figure S3).

### Combined treatment with Met and Prop reduces *in vivo* breast tumor growth affecting positively mice survival

**Figure 5:**
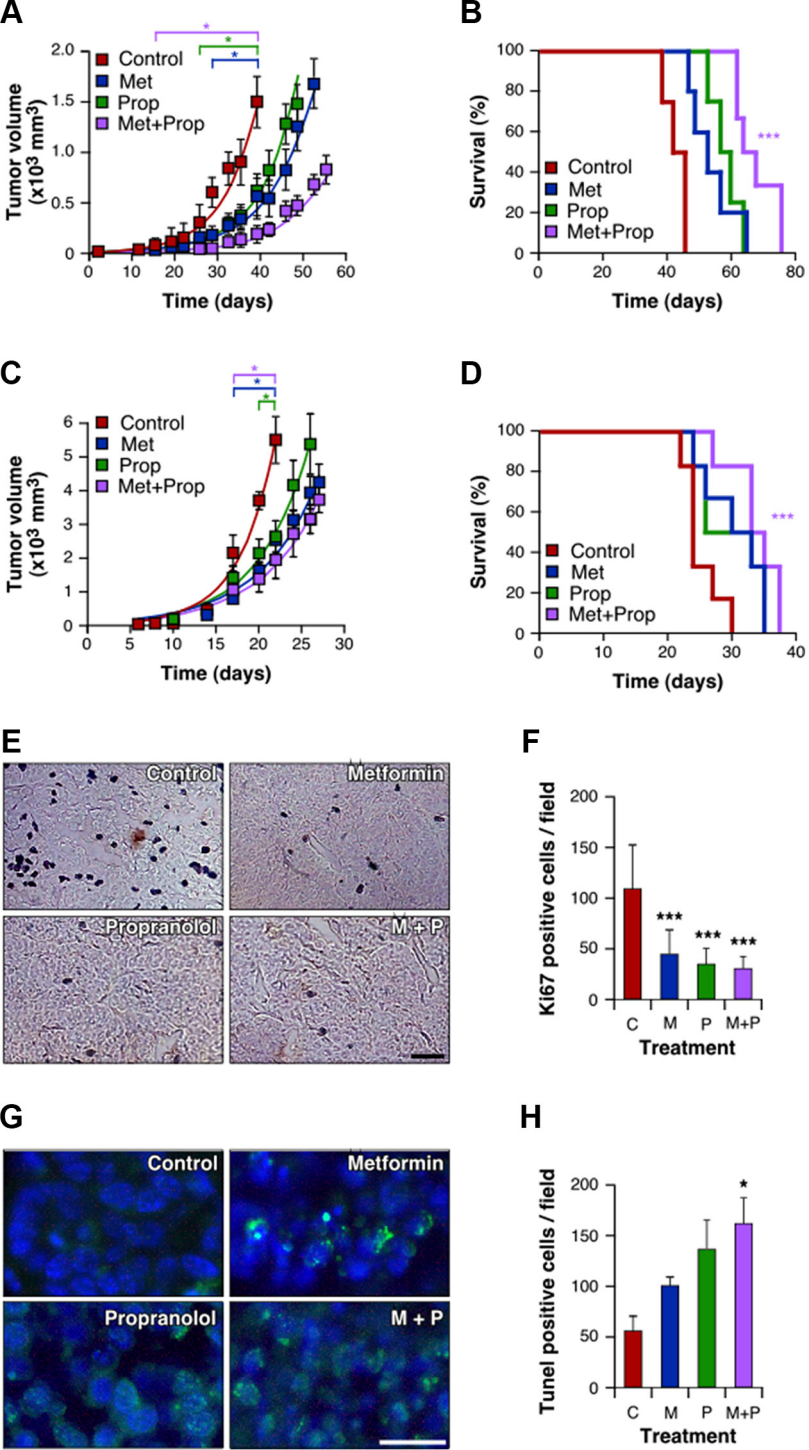
Tumor growth delay and improved survival of animals treated with Met and Prop Immunocompetent mice were orthotopically challenged with 4T1 cells (**A**, **B**) or M-406 tumor inolucum (**C**–**H**). Three days later Met (2g/l), Prop (25 mg/l) or both were added to the drinking water. The tumor size was measured biweekly with a calliper (**A**, **C**; *P* < 0.05 since the day indicated between square brackets). Survival was estimated for each group and data were statistically analyzed by long-rank Mantel-Cox test (**B**, **D**, *P* < 0.001). During exponential growth, tumors were collected and histological sections stained (**E**) and quantified (**F**) for Ki67 proliferative marker, or stained (**G**) and quantified (**H**) to determine apoptosis by TUNEL assay (scale bar = 50 μm).

To confirm our *in vitro* findings, we examined the effect of metronomic treatment with the drugs in two models of TNBC: 4T1 and M-406. Drug dosage was based on literature data and was associated with no toxicity. The choice was based in articles using Met or Prop to treat diabetes and hypertension respectively [25, 26]. Mice treated with Met and/or Prop developed tumors which showed a significant slower growth kinetics, as measured by the decreased tumor volume when compared to the control and the increased tumor doubling time (Figure 5A, 5C and Supplementary Figure S4A, S4B). This decrease in tumor growth also correlated with reduced proliferation associated with treatments (*P* < 0.001), as seen by immunostaining with Ki67 proliferation marker (Figure 5E, 5F). The combined treatment was more efficient in slowing down tumor growth (4T1: *P* < 0.05 from day 15; M-406: *P* < 0.05 from day 20; Figure 5A, 5C and Supplementary Figure S4A, S4B), which is related to a significant increase in apoptosis (*P* < 0.05; Figure 5G, 5H). Combined treatment was also associated with a significantly (*P* < 0.001) improved survival of mice bearing both 4T1 and M-406 tumors (Figure 5B, 5D). Interestingly, there were no signs of toxicity associated with treatments as seen by analysis of mice weight evolution (Supplementary Figure S4C, S4D) and general behavior (data not shown).

### Combination of Met and Prop reduces metastasis development

**Figure 6:**
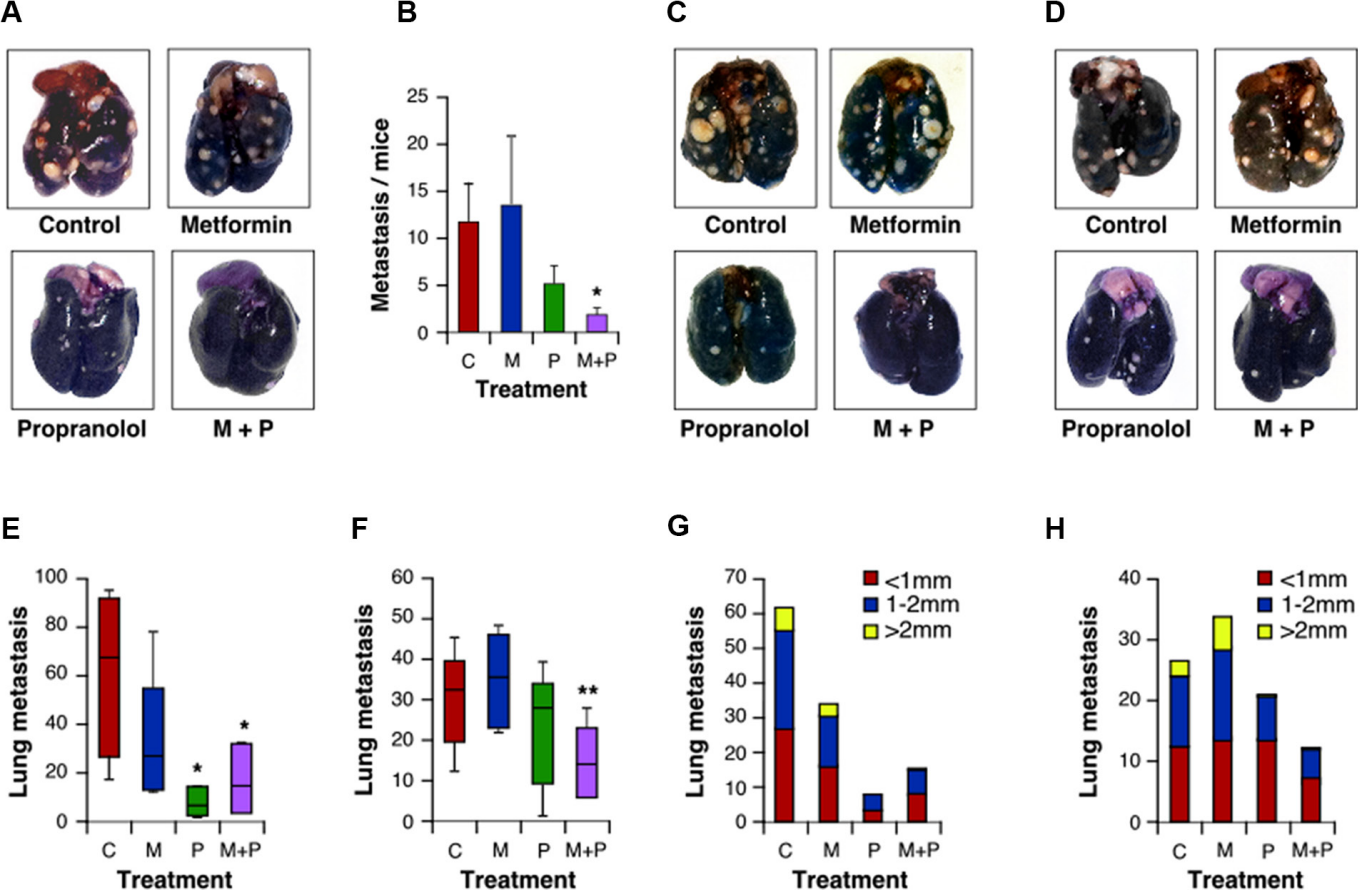
Combination of Met and Prop prevent development of metastasis Lungs from mice carrying M-406 tumors were observed at the time of death to determine the presence of spontaneous metastasis (**A**, **B**). Mice were i.v. injected with 4T1 cells (**C**) or M-406-derived cells (**D**). Fifteen days after injection lungs were stained with ink to allow metastasis quantification. Data are shown in a box and whisker plot and represented as median, first and third quartiles, and maximum and minimum of all data (**E**: 4T1 cells; **F**: M-406-derived cells). Metastatic nodes diameters were measured with a calliper (**G**: 4T1 cells; **H**: M-406-derived cells; C: control; M: Met (2 g/l); P:Prop (25 mg/l); M+P: Met+Prop; *n* = 7).

The examination of tumor-bearing animals, at the end of the tumorigenesis assays, indicated that the number of metastasis of M-406 cells to the lungs was significantly decreased (*P* < 0.05) upon treatment with Met and Prop (Figure 6A, 6B; Table 2). Nevertheless, there were no significant differences in metastasis number at the end of the experiment for 4T1 cells (Supplementary Figure S4E).

**Table 2:** Analysis of lungs collected from mice bearing M-406 breast tumors under the indicated treatments

Mice with metastasis		
Treatment	%	n^met^/n^tot^
*Control*	100	16/16
*Metformin*	84.21	16/19
*Propranolol*	72.22*	13/18
*Metformin + Propranolol*	58.33*	7/12

* Prop vs Control: *P* = 0.022448.

*M + P vs Control: *P* = 0.018521.

Significant differences obtained by Chi-square statistic test analysis are indicated. n^met^; number of mice with metastasis. n^tot^; total number of mice.

To further widen the analysis of the effect of treatments on lung colonization properties by breast cancer cells, we introduced M-406 or 4T1 cells intravenously in recipient mice. Consistent with our previous finding, we observed that breast cancer cells had impaired lung colonization ability when mice were under combined treatment (Figure 6C–6F), suggesting that treatments are, at least, affecting post-intravasation steps during metastasis process. Indeed, inspection of metastasis evinced a reduced size for metastatic nodules when mice were treated with Prop, either alone or in combination with Met (Figure 6G, 6H), suggesting also an effect of this β-blocker on the ability of cells to grow on lung epithelium.

## DISCUSSION

TNBC represent from 10 to 20% of breast cancer and is usually associated with poor prognosis and limited treatment options [27]. Cancer therapies currently applied cause significant side effects negatively impacting the patient's quality of life [28, 29]. Hence, there is an urgent need to develop clinically effective and well-tolerated new therapeutic approaches.

Met and Prop are being currently used to treat diabetes and cardiovascular illnesses, both conditions known as age-related diseases. Common cancer is also considered an aging-related condition, so the use of anti-aging repurposing drugs could be helpful to prevent or treat cancer [30–32]. During the last decade Met has been associated with a reduced incidence and severity of several types of cancer, and retrospective studies suggested a benefit of using ß-blockers for breast cancer patients [6–8, 33].

We report here that a combination of two repositioned drugs, namely Met and Prop, was able to slow down the growth of all the triple negative tumor cell lines and tumor-derived cells tested. They also were effective on the estrogen receptor (ER) positive MCF7 cells. Indeed, in all the cell types analyzed and regardless the method used, combination of Met+Prop showed a significantly stronger effect on cell growth than any individual drug. In fact, our *in vitro* data with 4T1 and MDA-MB-231 cells revealed that Met and Prop worked together in a synergistic manner blocking proliferation as it can be easily seen by the position of IC_50_ values falling below the additivity curve. Interestingly, the combination of these two drugs was effective not only to inhibit proliferation, but also to diminish the clonogenic capability and to increase apoptosis of the tumor cells.

In order to somehow mimic the metronomic administration of drugs under study, we performed proliferation assays in continuous presence of Met or Prop. It was evident that constant administration of low doses of Met and Prop had a stronger effect on cell proliferation than a short-term exposure, as seen by the drastic decrease (around 20 times for Met and 40 times for Prop) on IC_50_ value for these drugs when metronomically administered. This is not a special feature of these two drugs, but rather a consequence of the administration schedule. Indeed a stronger effect of topotecan and pazopanib was also recently described when supplied metronomically in breast cancer preclinical models [34]. To date, there is not clear correlation between *in vitro* Met effective antitumoral doses and the concentration of this drug observed in patient's plasma [35, 36]. Nevertheless, our observation of increased effectiveness (i.e. decrease on IC_50_ values) for both Met and Prop when administrated metronomically, together with their synergistic action, allow us to speculate that metronomic administration of this combined treatment could be effective at levels of Met as low as the ones found in patient's plasma.

Importantly, results from our *in vivo* experiments in TNBC models corroborate the *in vitro* efficacy of the combination. Animals receiving combined treatment were indeed those with a slower tumor growth, for both M-406 and 4T1 tumors. Moreover, Met+Prop treatment improved survival of tumor bearing mice, which also showed no signs of toxicity. This combinatorial treatment thus endows with two important features: effectiveness and lack of toxicity. Our data are in agreement with a recent publication that reported the effectiveness of combining Met and β-blocker atenolol in a mouse tumor model [37]. Nevertheless, it is worthwhile mentioning that the antitumor effect was achieved with atenolol doses that were quite higher than those of Prop used herein. This could be due to the action of propranolol on beta-2 adrenergic receptors (ADRB2). Interestingly, it was recently reported that Prop inhibits glucose metabolism of breast cancer cells through ADRB2-dependent posttranscriptional downregulation of hexokinase-2 [38]. It is also important to note that Prop is able to inhibit M2 macrophages polarization, triggered by activation of ADRB2 [39], an issue that could account for the reduced tumor growth in immunocompetent mice which, however, needs to be explored in more detail.

The antineoplastic activity of Met has attracted the attention of many researchers. It has been found that Met triggers cell cycle arrest at the G1 phase and induces apoptosis in a wide panel of breast cancer cell lines [40]. These effects are likely to be related to AMPK activation, enzyme that senses redox and metabolic imbalance [41]. Conversely to what was initially hypothesized by Warburg, it is now widely accepted that mitochondria are functional in a large majority of tumors [42]. The discovery that Met inhibits mitochondrial complex 1 activity in cancer cells opened new horizons for biguanides in the field of cancer metabolism. The mitochondrial effect is confirmed in our study by OCR abolition in breast cancer 4T1 cells and, consequently, by a severe reduction in mitochondria-driven ATP synthesis.

On the other hand, we showed here the anti-proliferative properties of Prop in various breast cancer cells, in agreement with our previous results in breast carcinoma *in vitro* and *in vivo* models [43]. We contributed to highlight the drug deleterious effects on mitochondrial network functions and dynamics in various cancer cells [44, 45], but Prop mechanism of action remained non-elucidated in breast cancer cells. Here, we showed that Prop induces apoptosis in various breast cancer cell lines. Moreover, we revealed that it disrupts bioenergetics in intact cells, and especially through the inhibition of respiration and ATP generation by mitochondria. Our results are consistent with a very recent study showing that atenolol inhibits the respiratory chain complex I in MDA-MB-436 and ZR-75-1 breast cancer cells [37].

We further showed the efficacy of combining Prop to low doses of Met to achieve a complete suppression of the mitochondrial bioenergetics. Prop may thus enhance the Met-mediated inhibition of mitochondrial-dependent metabolic intermediates required for cell growth and survival [16, 46]. Consistently, complete abolition of mitochondrial respiratory activity has been shown to prevent tumorigenesis in murine breast cancer models [47] and to inhibit the *in vivo* growth of human breast cancer cells [48]. The drop in mitochondrial oxidative phosphorylation rate in cells exposed to the Met+Prop combination led, in turn, to a noticeable increase in glycolysis. The metabolic reprogramming from mitochondrial respiration to aerobic glycolysis is generally described to support cancer cell proliferation and tumor growth [49]. Nevertheless, previous studies have demonstrated that Met can slow down cancer cell division in presence of abundant amounts of glucose and induce cancer cell death in deprived glucose conditions [16, 50]. By enhancing glycolysis to an extraordinarily high rate, the Met + Prop combination probably leads to glucose deprivation in tumor cell microenvironment. Combining these two stress-energy mimickers may thus first inhibit cell proliferation and ultimately produce metabolic synthetic lethality, as glucose levels decrease. Moreover, the rapid glucose processing in Met-treated cells has been suggested to result in the depletion of key glycolytic intermediates [51], effect that could be intensified by the combination with Prop. Indeed, it is important to note that all the observed effects of Met and Prop on mitochondrial-dependent metabolism could be associated with a putative inhibition of mitochondrial superoxide production as these drugs both inhibit mitochondrial complex I and propranolol display antioxidant effects [19].

Progression of cancer is a dynamic process that also requires the gain of invasive and migratory capabilities. A proof-of principle epidemiological pilot study highlighted that β-blockers reduce distant metastasis in breast cancer patients [52]. Recent studies showed that these drugs could decrease the pro-migratory effect of β-adrenergic receptors [53] and inhibit cell scattering by enhancing cell-cell adhesion [54] in breast cancer models. In the present study, we observed that the combination of Met + Prop altered breast cancer cell abilities to migrate and invade *in vitro*. Consistently, Met + Prop combination was highly efficient in preventing metastatic spreading *in vivo*, by diminishing the seeding capacity and the cell growth at the secondary tumor sites. This treatment feature is extremely important, as the main cause of mortality in patients with breast cancer is the development of metastasis and the scarcity of therapeutic options.

There is increasing evidence that metabolic plasticity is a major driver of cancer metastasis. While the aerobic glycolysis has long been thought to be linked to the metastatic potential of tumors, recent studies demonstrate that OXPHOS is indispensable for tumor migration and metastasis (extensively reviewed in [55]). Disseminating breast cancer cells indeed display increased levels of mitochondrial respiration [56]. The ability of Met + Prop combination to inhibit rapidly and efficiently the mitochondrial bioenergetics thus likely underlies the strong anti-metastatic properties of the treatment. Of note, this therapeutic strategy could especially be important to prevent breast cancer brain metastases, in which enhanced oxidative metabolism is associated with strongly increased tumor cell survival and proliferation [57].

Taking all together, our results indicate that the combination between Met and Prop is particularly effective in two different triple-negative, syngeneic breast cancer models, causing a significant metastasis-free prolonged survival with a favorable toxicity profile. This strongly suggests the possibility of using these repositioned drugs combination for the treatment of triple-negative metastatic breast cancer. As a matter of fact, it would be extremely interesting to test the benefits of such a combination when using as an adjuvant treatment after surgery in order to prevent metastasis development and/or recurrences or even as a perioperative therapy during the surgical resection of tumors.

## MATERIALS AND METHODS

### Cell culture

All cells were incubated at 37°C with 5% CO_2_. 4T1 cells were kindly provided by Dr. N. Zwirner (IBYME-CONICET). M-406 and M-234p derived-cells were obtained by disruption of tumors as described before [58]. These cells were cultured in RPMI supplemented with 10% fetal calf serum (FCS), penicillin (10 μg/ml), and streptomycin (100 μg/ml). MCF7 and MDA-MB-231 were kindly provided by Dr. G. Gil (CIQUIBIC-CONICET) and were maintained in DMEM supplemented with 10% FCS, penicillin (10 μg/ml) and streptomycin (100 μg/ml).

### Proliferation

For *in vitro* assessment of cell proliferation inhibition by Met (Metformin hydrochloride, Sigma-Aldrich) and Prop (Propranolol hydrochloride; Sigma-Aldrich) and their combination on breast cancer cells, 5 × 10^3^ cells/well were plated on 96-well culture plates. After attachment, different concentrations of Met and/or Prop were added and cells were allowed to grow for 24 hours. The number of living cells was estimated indirectly by tetrazolium salts reduction method (WST-1, Sigma Aldrich) as described by the manufacturer. The amount of formazan dye formed directly correlates to the number of metabolically active cells in the culture. Proliferation was expressed as the percentage of control untreated samples.

The concentration of drugs that decreased cell proliferation by 50% (IC_50_) as compared to controls was calculated using experimental data with the ED50plus v1.0 program. To determine IC_50_ values on metronomic treatment, cells were plated in 24-well plates (10^3^ cells/well) and allowed to attach. Cells were treated with Met or Prop continuously for 144 h in 0.1 ml of medium, adding fresh medium and drug every 24 h to mimic continuous metronomic treatment [59].

### Impedance measurements of cell proliferation

Impedance-based real time detection of cell proliferation and viability was done using the xCELLingence technology of the RTCA SP system (ACEA Biosciences, Ozyme, France). 4T1 or MDA-MB-231 breast cancer cells were seeded in E-Plate 96-well plates (5 × 10^3^ cells/well) for 24 hours, and were treated with metformin 1–7.5 mM alone, propranolol 10 μM alone, or their combinations. At cell seeding and after drug administration impedance changes were monitored every 5 minutes for 8 hours, and every 15 minutes for the rest of the experiment. Cell index (CI) values derived from the recorded impedance data, were normalized (NCI) using the RTCA Software 2.0 (ACEA Biosciences, Ozyme, France). Data of each single well were normalized to the first measurement after starting treatment using the equation NCI_(time)_ = CI_time_/CI_nml time_.

### Clonogenic efficiency

Cells (500/well) were plated on 6-well plates. After attachment, they were cultured in the presence of Met (5 mM) and/or Prop (5 μM) during 8 days. Photos of the clones were taken at different times and their size was estimated by measuring colonies diameters with the Image J software. After fixing the cells with formalin (4% PBS-buffered p-formaldehyde (Anedra)), colonies were stained with Giemsa to allow quantification.

### Apoptosis

After 24 hours of treatment with Met (5 mM) and/or Prop (5 μM), cells were collected, washed and stained with Annexin V-FITC (AP-Biotech) and Propidium Iiodide. Apoptotic rates were determined by flow cytometry.

### Migration

A wound was performed with a yellow tip on subconfluent 4T1 or MDA-MB-231 cells (time 0). Cellular motility was estimated by measuring closure of the initial wound. Photos were taken at the indicated times and quantification of healing was performed using the Image J software. Areas under the curve values were determined as described [60]. To analyze cellular motility in non-proliferative conditions, cells were put on media supplemented with 0,1% FCS overnight, and maintained under this condition during the whole assay.

### Invasion assay

*In vitro* cell invasion assays were performed in 10-mm diameter and 8 μm pore polycarbonate filter transwell plates (Millipore). Membranes were pre-coated with 20 μg of matrigel (BD Biosciences) on the upper surface, which formed a reconstituted basement membrane at 37°C. Cells (8 × 10^5^ in 100 μL media + 1% FBS) were seeded onto the upper well of the chamber, and the lower well was filled to the top (600 μL) with media + 10% FCS as chemoattractant. Treatments (Met 5 mM and/or Prop 5 μM) were added to both upper and lower media. After 24 hours incubation, cells were fixed in formalin and stained with Giemsa. Non-migrating cells were then carefully removed from the upper surface of the transwell with a wet cotton swab. Cells that had migrated to or invaded the bottom surface of the filter were counted. Six evenly spaced fields of cells were counted in each well using an inverted phase-contrast microscope.

### Real-time analysis of energetic metabolism

Multiparameter metabolic analysis of intact cells was performed in the Seahorse XF24 extracellular flux analyzer (Seahorse Bioscience, Billerica, MA, USA). 4T1 cells were seeded in XF24 V7 24-well plates (1.5 × 10^4^ cells/well) and incubated overnight at 37°C in 5 % CO_2_. Cells were then treated with metformin and/or propranolol for 4, 24 or 48 hours prior to the assay. To measure the glycolytic activity, cell culture medium was replaced for 1 h with minimal DMEM (0 mM glucose) supplemented with 2 mM glutamine and 1 mM sodium pyruvate, pH 7.4. Extracellular acidification rate (ECAR) was measured after sequential injections of glucose (10 mM), of the ATP synthase inhibitor oligomycin (1 μM), and of the glycolysis inhibitor 2-deoxyglucose (100 mM). To measure the mitochondrial activity, the same assay medium was used and supplemented with 10 mM glucose. Oxygen consumption rate (OCR) was analyzed under these basal conditions and after sequential injections of oligomycin (1 μM), of the electron transport chain uncoupler FCCP (1 μM) and of specific inhibitors of the mitochondrial respiratory chain antimycin A/rotenone (0.5 μM). To normalize OCR and ECAR data to cell number, 4T1 cells were simultaneously seeded and treated in a second multi-well plate. Cells were fixed with glutaraldehyde 1%, stained with violet crystal 0.1% (in methanol 20%), which was solubilized in DMSO to be analyzed with a microplate spectrophotometer.

### Animal experiments

Six to eight-week-old inbred BALB/c and CBi female mice were obtained from our breeding facilities. Animals were fed with commercial chow and water *ad libitum* and maintained in a 12 h light/dark cycle. The animals were treated in accordance to the Canadian Council on Animal Care guidelines. Tumor bearing mice were euthanized by CO_2_ exposure. CBi mice were described before [58].

To carry out 4T1 primary breast cancer tumorigenesis analysis, 5 × 10^3^ viable cells were resuspended in PBS (100 μl) and injected orthotopically into the fourth right mammary gland of the recipient mouse. For M-406 tumorigenesis, a tumor fragment of around 1 mm^3^ was orthotopically implanted in the fat pad, in the right mammary flank. Three days later, animals (*N* = 5–8/group) were distributed and treated as follows: *Control*: regular drinking water; *Met*: Met in drinking water (400 mg/kg BW/day); *Prop*: Prop in drinking water (7 mg/kg BW/day); *Met* × *Prop*: Met + Prop treatments combined. Primary tumor growth was analyzed by measuring tumor length (a) and width (b) with a caliper, and by calculating tumor volume (V) with the formula V = 0.4ab^2^. Fitting to exponential growth and tumor volume doubling times were calculated with the Prism 6 software. Metastatic dissemination of tumor cells was quantified *de visu* after sacrificing the mice and at the same time tumors were extracted, fixed in 4% formaldehyde, and embedded in paraffin for hematoxylin/eosin staining. To highlight metastatic nodes, lungs were stained as described later on.

To carry out direct lung colonization assays, either 5 × 10^4^ 4T1 cells or 2 × 10^5^ M-406 cells were resuspended in 100 μl PBS and injected into the lateral tail vein. After 2 wk, mice were euthanized, the chest cavity was exposed through a midline chest incision, the trachea cannulated with a 20-gauge needle, and lungs slowly inflated using 1 ml of India ink (Pelikan, 1:16 dilution in PBS). Lungs were then extracted, immersed in Fekete's solution (100 ml 70% ethanol (Cicarelli), 10 ml 4% formaldehyde (Anedra), and 5 ml 100% glacial acetic acid (Cicarelli)) to destain and metastatic nodules counted *de visu*.

### Immunostaining techniques

Tissues were extracted, fixed in 4% paraformaldehyde (Anedra), cut into 5- to 6-μm- thick sections, and stained with H&E. For immunohistochemical staining, paraffin-embedded sections were incubated with rabbit polyclonal antibody to Ki67 (Leica Biosystems, 1:50 dilution). After overnight incubation, tissue slides were rinsed with PBS, incubated for 1 hour at room temperature with Vectastain Elite ABC kit (Vector Laboratories, USA), developed with 3,3´-diaminobenzidine (Sigma) and counterstained with hematoxilin. Quantification was done in a blind fashion by counting positive cells in 10 fields (400X). Apoptotic cells were detected with the TUNEL-based In Situ Cell Detection Kit (In situ cell death fluorescein, Sigma): Sections were deparaffinized, hydrated, and digested with proteinase K (Dako) for 30 min at 37°C and then subjected to the TUNEL reaction according to the manufacturer's instructions; TUNEL-positive cells were visually scored with a standard immunofluorescence microscope (CTR600, Leica) and counted blindly in 8 fields (400X) chosen at random.

### Statistics

Data obtained in lab experiments were analyzed using ANOVA and Tukey-Kramer Multiple Comparison tests, Kruskal-Wallis and Dunn's post-test, and Log-rank test were used to examine the differences between groups with GraphPad Prism version 3.0 (GraphPad Software, San Diego, CA). Unless otherwise indicated, results are expressed as mean ± sem of three independent experiments. *P* values lower than 0.05 were considered statistically significant.

## SUPPLEMENTARY MATERIALS FIGURES AND TABLES


