# Supplementary material for: Metformin and propranolol combination prevents cancer progression and metastasis in different breast cancer models
Source: Oncotarget. 2016 Dec 1;8(2):2874–89. doi: 10.18632/oncotarget.13760 (PMC5356849; doi:10.18632/oncotarget.13760)
Supplement: Supplementary file 1 [file oncotarget-08-2874-s001.pdf]

Metformin and propranolol combination prevents cancer progression and metastasis in different breast cancer models

Supplementary Materials

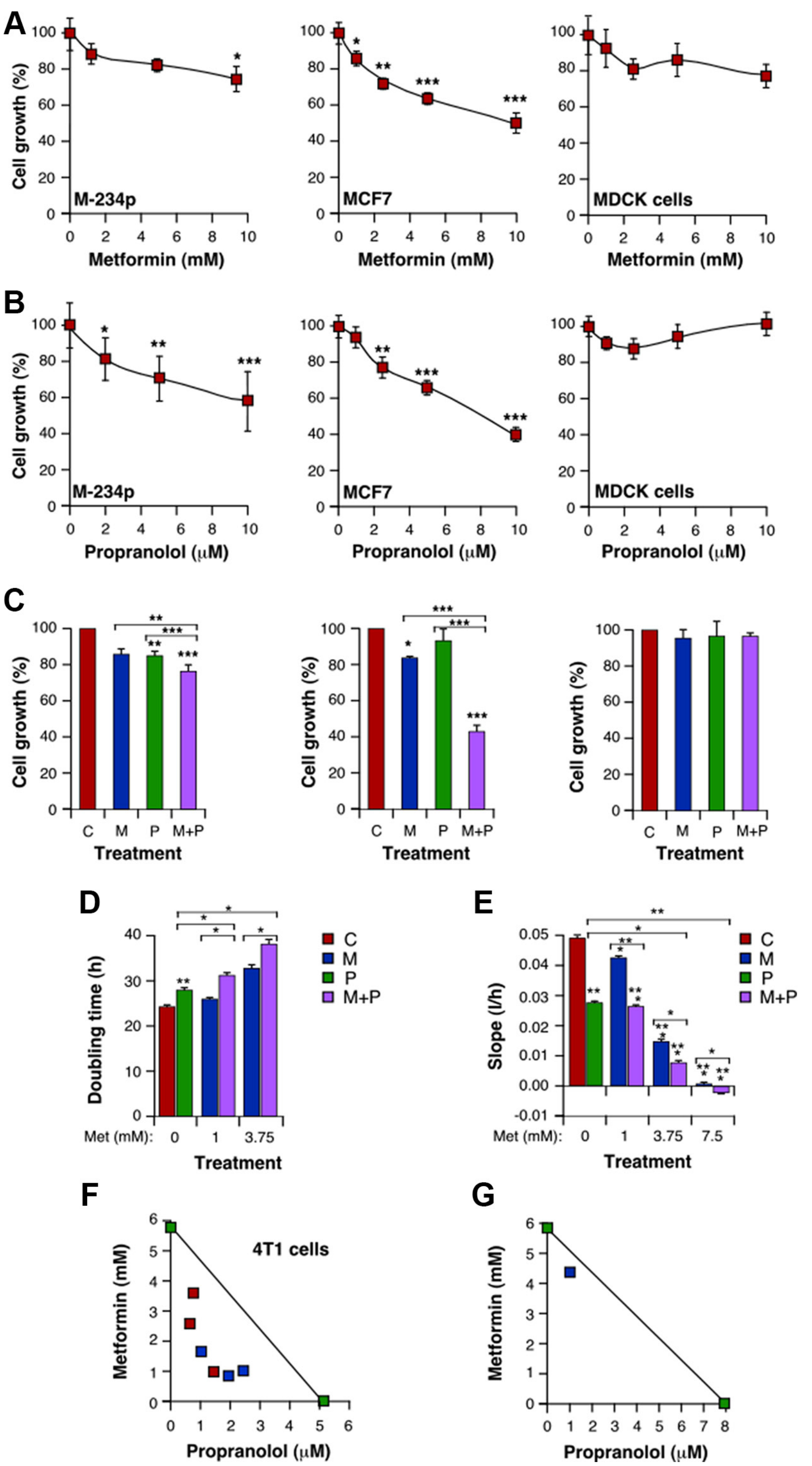

Supplementary Figure S1: Met and Prop effect on breast tumor cells viability. Cells were cultured in the presence of the indicated doses of Met (**A**) or Prop (**B**) during 24 hours. The number of metabolically active cells was estimated by tetrazolium salts reduction method (n = 3). (**C**) M-234p-derived cells (left panel), MCF7 (middle panel), and MDCK cells (right panel) were treated for

24 hours with Met (M), Prop (P) or a combination of them (M+P) and living cells were estimated as before (n = 3; for combined treatment Met concentration was 1 mM for M-234p and MCF7 and 5 mM for MDCK cells, and Prop concentration was 1 µM for M-234p and MCF7 and 5 µM for MDCK cells). (**D**, **E**) Doubling time calculated during an exponential phase of cell growth, i.e. from 24 h after seeding to 80 h, in real time cellular analysis profiling of 4T1 cells. (E) Slope calculated over 48 h of treatment, from 40 h to 88 h post-seeding. (**F**, **G**) Isobolograms were built up for 4T1 (F) and MDA-MB-231 cells (G) for analysis of synergism. Green squares indicate IC50 value for each drug; red squares indicate IC50 values for Prop in the presence of the corresponding dose of Met; blue squares indicate IC50 values for Met in the presence of the corresponding values of Prop. (C: control; M: Met; P:Prop; M+P: Met +Prop; n = 3).

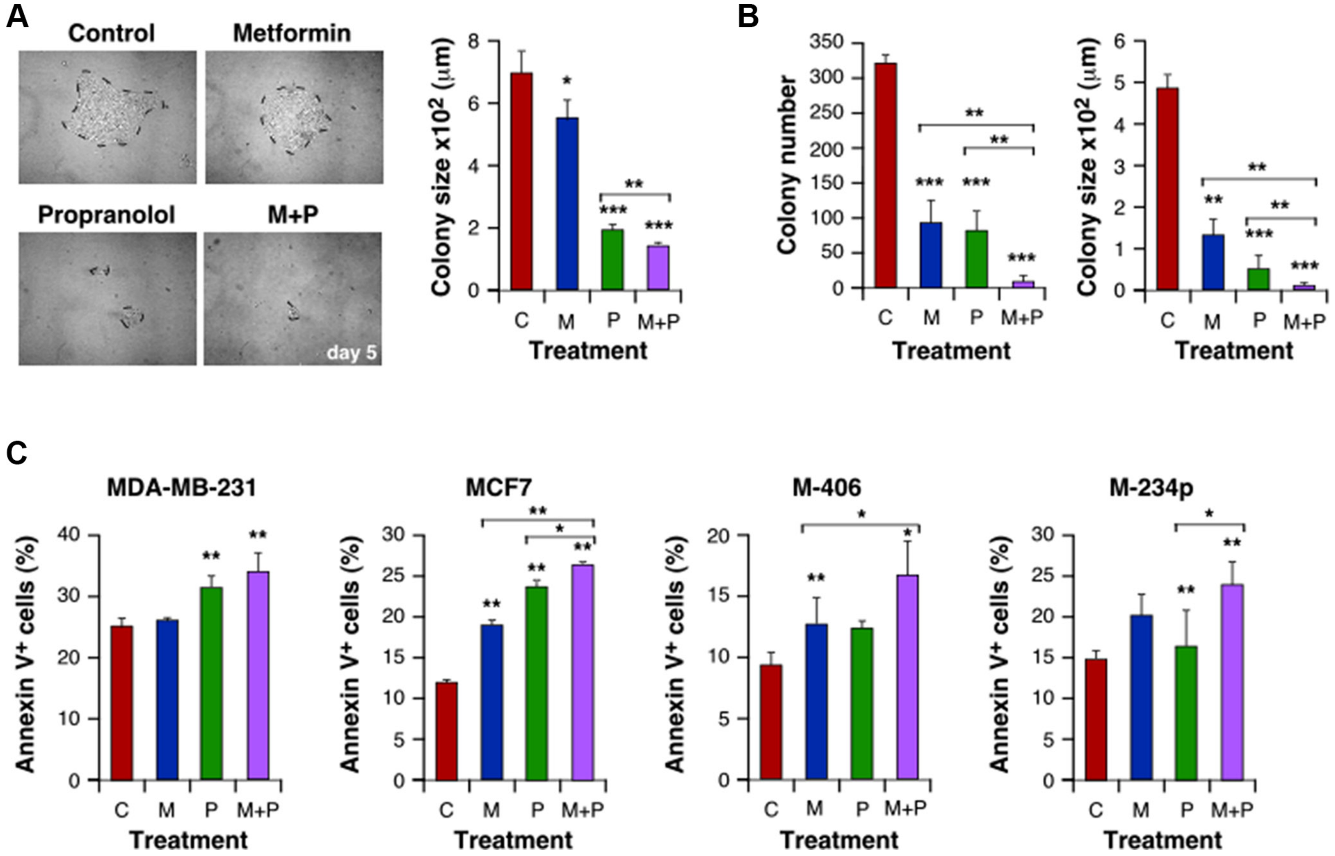

Supplementary Figure S2: Met and Prop affect the clonogenic behaviour and trigger apoptosis in breast cancer cells. 4T1 clones photos were taken at day 5 after seeding and their size was estimated (**A**). (**B**) 8 days after seeding, MDA-MB-231 clones were counted (left panel) and colony size was estimated by measuring colonies diameters with the Image J software (right panel). (**C**) Quantification of the percentage of Annexin V$^+$ apoptotic indicated cells. (M: Met 5 mM, P: Prop 5 µM, M+P: Met+Prop; n = 3).

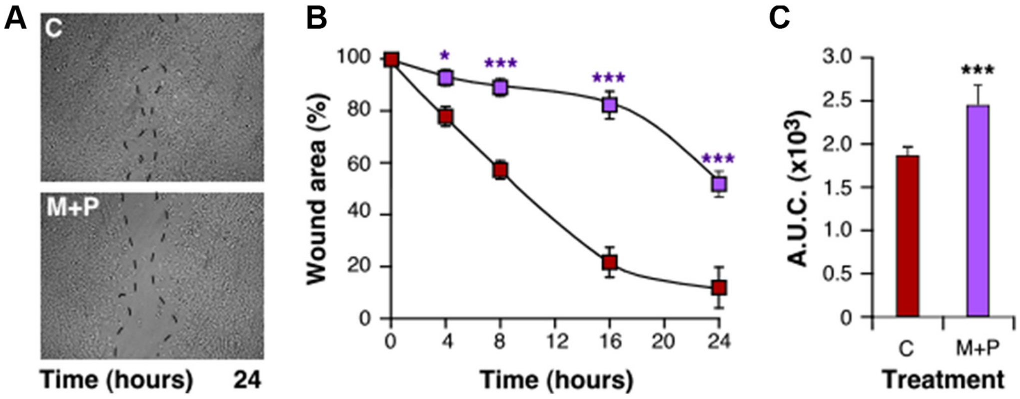

Supplementary Figure S3: Met and Prop affect metastatic-related events *in vitro*. After attachment, 4T1 cells were maintained overnight at starving conditions (0.1% fetal bovine serum). Wound healing assay was performed in starving condition as described in Materials and Methods. Cellular motility was estimated by measuring closure of the initial wound. Photos were taken at the indicated times (**A**). Quantification of healing was performed using the Image J software (**B**) and the area under the curve (A.U.C.) was calculated (**C**) M + P = Met 5 mM + Prop 5 μM, *n* = 3.

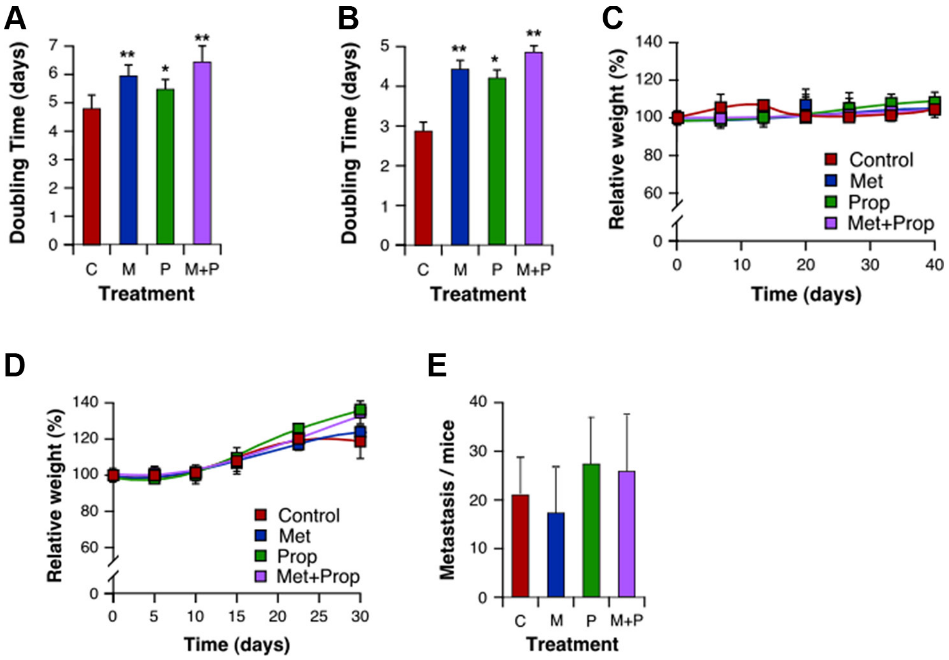

Supplementary Figure S4: Tumor growth delay and improved survival of animals treated with Met and Prop. Doubling time for 4T1 (**A**) and M-406 (**B**) tumors were estimated from the exponential curves showed in Figure 5. BALB/c (**C**) and CBi (**D**) mice carrying tumors were periodically weighted. (**E**) Quantification of spontaneous metastasis for mice with 4T1 tumors.
